# Delayed Presentation of Patients with Hip Fractures during the COVID-19 “Stay-at-Home” Order in the Southmost Region of the United States

**DOI:** 10.1155/2021/8822004

**Published:** 2021-02-16

**Authors:** Michael Serra-Torres, Raul Barreda, David Weaver, Annelyn Torres-Reveron

**Affiliations:** ^1^DHR Health Orthopedic Institute, Edinburg, TX 78539, USA; ^2^DHR Health Institute for Research and Development, Edinburg, TX 78539, USA; ^3^DHR Health Surgery Institute, McAllen, TX 78504, USA

## Abstract

To evaluate the effects of COVID-19 and stay-at-home orders in traumatic hip fractures presentation, we conducted a retrospective chart review cohort study from March 13 to June 13 in 2020 compared to 2019 from a single-hospital Trauma Level 2 Center. Males and females, 18 years of age and older presenting with a diagnosis of displaced or nondisplaced, intracapsular, or extracapsular hip fracture, underwent standard of care—comparative analysis of the patient's characteristics and clinical outcomes. The primary study outcomes included age, sex, ethnicity, and body mass index, the onset of injury, date of arrival, payer, the primary type of injury and comorbidities, mechanism of injury, treatment received, postoperative complications, days in an intensive care unit (ICU), discharge disposition, pre- and postinjury functional status, and COVID-19 test. Age, sex, ethnicity, and body mass index were similar in the patients in 2019 compared to 2020. The patients' average age was 76 years old, 80% reported Hispanic ethnicity, and 63% of the patients were females. Most injuries (90%) occurred due to falls. On average, patients in 2020 presented 4.8 days after the injury onset as compared to 0.7 days in 2019 (*p* < 0.05). There was an increase in displaced fractures in 2019 compared to 2020 and an increase in patients' disposition into rehabilitation facilities compared to skilled nursing facilities. Despite the delay in presentation, length of stay, days in the ICU, or functional outcomes of the patients were not affected. Although the patients showed a delayed presentation after hip fracture, this does not appear to significantly interfere with the short-term or the 6-month mortality outcomes of the patients, suggesting the possibility of guided delayed care during times of national emergency and increased strain in hospital resources.

## 1. Introduction

The COVID-19 pandemic has introduced many challenges to healthcare systems around the world, presenting added changes, pressures, and strains at all levels of the system, including but not limited to doctor–patient relationships and health organization resources [1, 2]. The pandemic has, in turn, produced several changes in the patient's behavior and clinical outcomes. Nevertheless, these changes have provided us the opportunity to examine variations in clinical presentations that would not have been possible under normal circumstances. On March 13, 2020, the State of Texas declared a state of emergency, shortly followed (March 22) by an order for the postponement of elective surgical procedures anticipating an increase in COVID-19-infected patients requiring usage of hospital resources. On April 2, the State entered into a stay-at-home order that extended until April 30. On May 1, the State started a phase one reopening, as recommended by the Department of State Health Services [3]. On May 18, Texas expanded to a phase two reopening plan, allowing for most of the economy to resume operations with some restrictions on their capacity. The sum of all these orders created a decrease in the mobility of the general public, resulting in a reduction of all traumatic injuries presenting at our hospital emergency service, including hip fractures. Changes in population behavior during the COVID-19 restrictions have given us an inside perspective on the impact of states of emergency in traumatic hip fractures.

Most hip fractures occur in the elderly population, with 90% occurring after the age of 50 years and 52% occurring after the age of 80 years [4]. Ninety percent of hip fractures occur due to falls from a standing height or less [5, 6]. Women experience 75% of all hip fractures, mainly due to the increased frequency of osteoporosis [7]. The State of Texas has the highest number across the United States for older adults who fall, reported at 33.9% [8]. Injuries due to falls represent a cost of 1.6 billion in Medicare expenses for Texas [9] and 29 billion for the United States annually [10].

Hip fractures can be categorized using multiple classifications. In this study, we classified fractures by displacement (displaced versus nondisplaced) and anatomic sites (femoral neck, intertrochanteric/subtrochanteric) since this classification guides the surgical options. Clinical guidelines recommend immediately surgical repair of hip fractures within 24 hours, preferably, or as soon as medically stable, but avoiding a delay in surgery beyond 72 hours [11–13]. However, there is still controversy in the literature regarding the definition of a delayed intervention in hip fracture repairs [14]. Mortality appears to decrease when patients are intervened within the 72 hours following the injury [15]. On the other hand, two large studies that adjusted for demographic characteristics and comorbidities reported that mortality rates are not affected by a delay in the surgical intervention of more than 120 hours [11–13]. Based on previous findings, we are defining a delayed presentation of hip fracture as any presentation for more than four days (96 hours).

The purpose of this study was to evaluate the effects of COVID-19 and stay-at-home orders in traumatic hip fractures presenting at a functioning Level 1 Trauma center in Texas. It was hypothesized that the behavior of patients seeking care during the COVID-19 pandemic has been altered by the governmental regulations with possible changes to the mechanism of injury, time of presentation, and outcomes of treatment. While other hospitals in the region entered into the diversion of patients, our hospital system continued to receive all types of patients. We were the only center able to allocate 200 beds for patients arriving with COVID-19 symptoms. Therefore, our orthopedic department continued uninterruptedly caring for all patients, including those being transferred from other hospitals into our hospital, regardless of their COVID-19 status. In light of this, the hospital implemented treatment algorithms designed to deliver timely treatment to patients while protecting the medical personnel in the emergency room and the operating rooms. Understanding how government-imposed restrictions affect clinical outcomes may provide a working framework for optimal healthcare delivery during state and national emergencies.

## 2. Methods

### 2.1. Study Design and Setting

This is a retrospective chart review cohort study of patients from a single hospital, Level 2 Trauma Center in South Texas. The Institutional Review Board approved this study, and it conformed to the Declaration of Helsinki and the US Federal Policy for the Protection of Humans Subjects. A full waiver of authorization under the Health Insurance Portability and Accountability Act (HIPAA, 1996) was submitted by the study team and approved by the Institutional Review Board to conduct this retrospective study. The retrospective period of chart review was set from March 13, 2020, to June 13, 2020. We used March 13, 2020, as the start because on that day, the state government issued the emergency order and up to three months after. This period includes data for the four weeks following phase two reopening of the State, allowing for a possible return to normal behavior and mobility in the population. To compare, we used the data extracted from the same period in 2019.

### 2.2. Subjects

Male and female subjects 18 years and older with a diagnosis of the displaced or nondisplaced femoral neck and intertrochanteric/subtrochanteric fractures with all its modifiers (ICD10: S72.0, S72.1, and S72.2) were eligible for the study. The patients were excluded from the study if they presented with a fracture already treated at another institution (referred to a rehabilitation hospital), if the onset of the injury or presentation was outside the study period, for the repair of nonunion fractures, or lack of documentation for the onset of the injury.

### 2.3. Variables

The demographic variables included were age, sex, ethnicity, body mass index, date of injury arrival (presentation), date of arrival, presentation site, health insurance, or self-pay. From the medical record, we obtained the onset of the injury, the primary diagnosis and secondary diagnosis, the mechanism of injury, treatment received and date, postoperative complications (i.e., respiratory failure, hypotension, anemia, infection, deep vein thrombosis, and hemorrhage), intensive care unit (ICU) usage, comorbidities and discharge disposition, pre- and postinjury functional level, and the COVID-19 test results, for patients who received it in 2020. The delay in the presentation was calculated from the date reported by the patient, when the injury occurred, to the date of arrival to the hospital (emergency room or clinic). The delay in surgery was calculated from the date of arrival to the date of surgery, as reported in the medical record. If surgical treatment was delayed due to medical optimization needed (i.e., coagulation), it was also noted during the data collection process.

To determine whether the delay in presentation represented a difference in functionality for the patients, we quantified the presurgical and postsurgical functionality level of patients into three main categories: walking with aid (walker, crutches, and rollator), bed-bound (nonambulating, including those that use a wheelchair for transportation or stand for transfer only), and walking independently. The functional level received a categorical score of zero (0) for bed-bound, one (1) for walking with aid, and two (2) for walking independently. To calculate the change in functionality, we subtracted the value on the level of functionality at discharge from that of the admission. A zero indicates no change in function from preinjury to postinjury, a minus 1 (−1) indicates a decrease in function (e.g., from walking independently to walking with aid), and a minus two (−2) indicates a decrease in functionality of two levels (e.g., from walking independently to bed-bound).

### 2.4. Data Source/Measurements

The hospital system maintains a trauma databank as part of the trauma quality improvement program from the American College of Surgeons. The trauma databank is populated by trained nurses for this task. Patients' meeting inclusion criteria from 2019 were identified via an electronic report from the hospital trauma databank. For 2020, we requested a report from the business intelligence department in the hospital for the three months' period under review, using the ICD-10 codes reported above. Once the list of patients and the corresponding medical record number were obtained, the same variables for all patients were extracted. All the presented information was part of the subject's standard of care, as documented in their medical record or the trauma data bank, and there was no intervention or variable collected directly from the patient. To maintain quality and consistency in the data extraction process, the same trauma data analyst extracted patient's additional information not present in the trauma databank “record from 2019” and 2020. Inclusion and exclusion criteria were verified by the orthopedic trauma surgeon before the patient was included in the analysis. After six months of the event, the patient's mortality was verified by their recorded attendance to follow-up visits or by notes from our hospital call center in charge of following up patient's status via phone calls.

### 2.5. Statistical Methods

Descriptive statistics were used for the entire study population and subdivided by year. Frequencies and column percentages were used to summarize categorical variables. The normal distribution of continuous variables was measured using the Shapiro–Wilk goodness-of-fit test. Nonnormally distributed variables were analyzed using the Wilcoxon test, and normally distributed variables were analyzed using the Student *t*-test for independent samples. Chi-square or Fisher's exact test was used for categorical variables. Multinomial regression analyses were used to explore the changes in function across injury types. The statistical analyses were two-sided and conducted using JMP 15.0 (SAS Institute, Inc., Carry, NC, USA). The significance was set at *p* < 0.05.

## 3. Results

### 3.1. Participants

From March 13, 2020, to June 13, 2020, 41 patients met the inclusion criteria. Six patients were excluded for the following reasons: (1) a visit related to a nonunion treatment, (2) a visit associated with the removal of the implant, (3) patient unable to report the onset of injury due to a history of multiple falls, (4) patient left against medical advice from the emergency room, (5) patient requested a transfer to a different institution, and (6) patient with rule-out treatment, with negative results. Data from 2019 was extracted from the trauma databank at our institution, and all encounters have already been reviewed and verified by qualified trauma data analysis. During the same period for 2019, 45 records were reported to the trauma databank. Four patients' records were eliminated because these were transferred to our institution with fractures already treated; forty-one records met the inclusion criteria in 2019.

### 3.2. Demographic Characteristics

Table 1 presents the demographic characteristics of patients per year. There was a female's predominance in the cohort of patients for both years with no difference in the gender distribution between years. The majority of the patients in both years (>80%) self-reported Hispanic ethnicity, which represents the demographic distribution of our region. The average age for the patient's cohort was 76.4 ± 14.9 years, and the mean age for both years was very similar. The body mass index was also similar between the 2019 and 2020 cohorts. There was no statistical difference in the demographic characteristics of patients between years (all *p* > 0.05).

### 3.3. Outcome Data

Table 2 presents the categorical clinical characteristics of the patients. The modes of injury for the hip fractures were in the majority (>90%) due to falls of less than a meter height (e.g., transferring from bed to commode) for both years (*X*^2^ = 1.7, d.f. = 2, *p* > 0.05). Similarly, 90% of the patients presented directly to the emergency room, with an average of 9% of patients going directly to clinics (*X*^2^ = 0.674, d.f. = 1,*p* > 0.05). The types of injuries that the patients presented upon arrival were different across years (*X*^2^ = 18.76, d.f. = 3, *p* < 0.01), with displaced fractures more frequently presented in 2019 than in 2020. Displaced intertrochanteric hip fractures were more frequent in 2019 than in 2020 compared to nondisplaced intertrochanteric, OR = 22.28 (95% CI, 2.12 to 233.85). Similarly, displaced femoral neck hip fractures were also more frequent in 2019 than in 2020 compared to the nondisplaced femoral neck, OR = 15.37 (95% CI, 2.37 to 99.56). The majority of the patients (>70%) had Medicare as the principal payer, which was predicted from the cohort's age range. We observed a significant difference in the payer between years (*X*^2^ = 8.69, d.f. = 3, *p* < 0.05). There was an increased odds ratio for the patients having Medicare instead of private/other insurance as a payer in 2019 compared to 2020: OR = 3.48 (95% CI, 0.5 to 24.06). The disposition of patients varied between 2019 and 2020 (*X*^2^ = 12.61, d.f. = 4, *p* < 0.05). While more than half of the patients were discharged to rehabilitation facilities during both years, an increased proportion of patients were discharged to skilled nursing facilities in 2019 compared to 2020 (OR: 8.88 (95% CI, 1.42 to 55.45). Only one patient died in 2020 (cardiopulmonary arrest in a 92-year-old male) and none in 2019. The proportion of patients with postsurgical complications was not different between years (*X*^2^ = 2.819, d.f. = 5, *p* > 0.05). The mortality of the patients at 6 months after the presentation was evaluated. Four patients were lost at follow-up: two in 2019 and two in 2020. There was a nonsignificant increase in mortality for 2020 at 17.1% as compared to 2019 at 9.8% (*X*^2^ = 0.969, d.f. = 1, *p* > 0.05). Despite the lack of significant difference, the odds of mortality at 6 months in 2020 compared to 2019 were moderately higher: OR = 1.94 (95% CI, 0.542 to 6.53). When only the patients with a delayed presentation were considered, only one patient from the 2020 cohort died and none from 2019.

### 3.4. Main Results

Table 3 presents the quantitative variables related to their inpatient period. The average time from injury onset to presentation in 2020 was 7.5 times higher compared to 2019 (0.7 days in 2019 vs. 4.8 days in 2020). Patients in 2020 presented to the hospital on average, four days later than in 2019. The delay in the presentation was significantly different between years (*Z* = 2.13, *p* < 0.01). The average among only those patients with the delayed presentation was 8.33 (range: 7 to 11) days in 2019 compared to 20.87 (range: 4 to 55) days in 2020. In 2019, only three (7.31%) patients showed up to the hospital with a delay larger than four days in 2019; in 2020, eight (22.85%) patients had a delayed presentation. Despite the significant delay in presentation between years, the time to surgical care was similar (*Z* = 1.41, *p* > 0.05). There was no difference between years regarding the total hospital length of stay (*Z* = 1.09, *p* > 0.05), the number of days spent in the ICU (*Z* = 1.43, *p* > 0.05), and the number and type of comorbidities (*Z* = −0.59, *p* > 0.05). The most frequently observed comorbidities were type 2 diabetes and hypertension.

**Figure 1:**
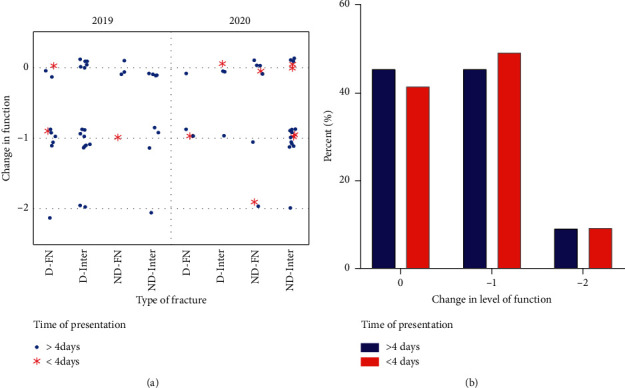
Changes in function resulting from hip fractures. (a) Comparison of the categorical change in function for each type of fractures. Change in function was assigned a value based on the mobility capacity before fracture and after fracture. A zero indicates no change from pre- to postfracture; negative one indicates a one-level decrease in function (i.e., from previously walking to using aid to walk); and negative two indicates a two-level decrease in function (i.e., from previously walking to being bedridden). No significant differences in functionality were observed between years. (b) The percent of patients within each categorical change in functionality was not affected by the delay in presentation (data collapsed across years). D = displaced, ND = nondisplaced, FN = femoral neck, and Inter. = intertrochanteric.

We explored the change in functionality within each type of fracture and presentation delay. Neither the type of fracture nor the delay in presentation affected the change in functionality across the years (Figure 1(a)). The categorical decrease function is maintained across both years and is not affected by the delay in presentation (Figure 1(b)). The percentage of patients that maintained the same level of function (zero category) was 45% and 41% in nondelayed compared to delayed presentation, respectively. Similarly, 45% and 49% of patients decreased one level of functionality (−1 category) in 2019 and 2020, respectively. Less than 10% of the patients decreased two levels of functionality (−2 category) regardless of delayed presentation.

In 2020, 8 patients had a delayed presentation to our institution. Qualitative analysis of the reasons for the delay in seeking care in 2020, as documented in the chart, revealed that two patients did not seek immediate care because they were afraid of COVID-19 contagion; one patient had an injury out of the country. One patient tried avoiding the emergency room due to COVID-19 and visited their primary care physician (PCP). Two patients had delayed diagnosis (X-ray) before the final diagnosis by advanced imaging (MRI and CT), and both were nondisplaced femoral neck fractures. Finally, two patients were bed-bound prior to the injury, and their respective families brought them to the hospital only after symptoms did not resolve for more than a week. On the other hand, in 2019, three patients had delayed presentation: one patient had a missed diagnosis of the fracture, one had a delay in workup at PCP, and for the third one, the reason for the delay could not be found in the chart.

### 3.5. COVID-19 Testing

From the 35 patients included in 2020, twenty patients had the COVID-19 test done. From May 28, 2020, to June 15, 2020, all patients received a COVID-19 test (13 patients). Before that, the test was done only upon the request of the orthopedic surgeon and depending on the test kit availability. All tested patients were negative for SARS-CoV-2. At the 6-month follow-up, one patient from the 2020 cohort resulted positive for COVID-19 shortly after the hip fracture and died due to the virus. This patient was not a delayed presentation.

## 4. Discussion

A delay in the presentation following hip fractures in the elderly population of South Texas during the COVID-19 pandemic did not significantly impact the length of stay, postsurgical complications, or functional outcomes compared to a similar demographic population with hip fractures during the same period of the last year. The delay in the presentation was significantly influenced by the absence or presence of displaced fractures. A lower number of displaced fractures were observed in 2020 compared to 2019. Displaced fractures are prone to increased complications and decreased patients' quality of life compared to nondisplaced fractures [16]. This could, in part, explain the incidence of nondisplaced fractures with a late presentation. Avoidance behavior, such as going to the PCP instead of the emergency room or family members expecting symptoms resolution, was observed. Our results align with a previous study using data from the American College of Surgeons, indicating that a surgical delay for pathological hip fractures was not associated with increased complications [17].

Delaying medical care, or otherwise known as medical care avoidance, is a multifactorial process. The three main categories that explain this behavior are (1) unfavorable evaluations, (2) low perceived needs, and (3) barriers to medical care, such as high cost and time constraints [18]. Unfavorable assessments are influenced by affective behavior as presented by Taber et al.; fear of bad news and unspecified fear, in general, were two of the factors documented in her work. While medical insurance was not a factor in the current study that statistically explained the delay, the lower perceived need, fear of bad news, a saturated medical system, and fear of COVID-19 contagion seem to have played a role in explaining patient's presentation. Additional studies in our hospital and other facilities across the nation are needed to understand the behavioral patterns in light of the pandemic and how it affects outcomes.

The current pandemic presents a significant strain on hospital resources and a possible increased risk of contagion for patients. With future emergencies leading to an overloaded system, it might become necessary to stratify care by weighing the risks and benefits of early/delayed intervention versus exposure. Our study appears to suggest that a possible delay, specifically in the treatment of nondisplaced hip fractures of an average of three weeks, does not result in an adverse effect on early outcomes. Furthermore, during emergencies, when the medical system is saturated, such as during the COVID-19 pandemic, the patient could be triaged at other clinical care facilities and brought into the hospital with decreased urgency (guided delayed treatment) to reduce exposure to external risk factors such as high contagious infectious diseases with increased mortality. We strongly believe that the clinical guidelines for surgical repair within the shortest time frame should always be followed. However, the possibility of guided delay during emergencies will require further research to measure feasibility and acceptability by patients and health care providers.

We observed an increase in rehabilitation disposition as compared to skilled nursing facilities in 2020. The most reasonable explanation for this observation results from the increased risks of COVID-19 infections and mortality in nursing homes that have been observed across the nation [19]. Thus, in times of emergency, the patient's postsurgical disposition might need to be weighed by both the benefit to the patient functionality and preventive measures to preserve optimal health. Discharge disposition could also explain the marginal increase in the length of stay in 2020 since some patients had to wait for placement at the rehabilitation hospital.

Cultural differences are well known to influence the type of care that is given to the elderly. The majority of the patients in this study were of Mexican-American descent due to the close proximity that our hospital has with the northern border of Mexico. When faced with a hip fracture diagnosis, Mexican-Americans prefer informal caregivers (family members or friends), rather than formal care in comparison to non-Latino whites [20]. Yet, Mexican-American elders are more functionally impaired than their non-Latino whites' counterparts [21]. Crist and Speaks [22] have documented that within the Mexican-American cultural norm, familism is strong: the care of the elderly is the responsibility of the family. During the stay-at-home order to reduce COVID-19 spread, there was an increased number of family members available to care for the elderly in the family nucleus. We presume that this availability resulted in increased supervising and assistance with activities of daily living to the senior members. An increased number of helping hands within the family structure may lead to a reduction in the risk factors for falls [23] or in the severity of the falls that occurred. This factor may partially explain the general decrease in the presentation of trauma arriving at our emergency room due to the reduction of exposure and potential severity of the fall.

In other parts of the world, significantly impacted by the COVID-19 pandemic, orthopedic surgeries have continued due to the essential nature of this service. Orthopedic surgeons and other surgeons in charge of trauma services have the imminent urgency to take care of the patients, with the increased susceptibility to contagious diseases of any type [24]. For example, in China, 26 orthopedic surgeons tested positive for COVID-19, with 80% of these exposures in the wards [25]. On the other hand, patients' undergoing surgical interventions due to traumatic injuries have poorer outcomes if they have active COVID-19 infections. This is confirmed by independent reports from New York [24], United Kingdom [26], France [27], and China [28]. While we only had one patient who resulted positive for COVID-19 shortly after surgery, the delay in surgical intervention in light of poorer outcomes in patients with active COVID-19 deserves additional attention at the national and multinational levels.

### 4.1. Study Limitations

One of the limitations of our study is the lack of long-term outcomes for the patients. The best indicator of outcome for hip fractures is mortality within a year [29]. However, the assessment of mortality at 6 months did not show a significant difference between 2019 and 2020. It still needs to be determined if the same outcome remains true at one year after fracture. We did not assess whether the delay in the presentation of care resulted in increased medical costs, family burden, or psychological stress. These are areas that could be explored in light of the multiple changes that have been implemented at hospitals in response to the pandemic. The sample size of the study was limited by the number of presentations within the selected time frame. As with any retrospective study, there was the confounding factor of information bias. A rigorous protocol for data extraction was uniformly followed for both years and verified by the orthopedic surgeon. As mentioned before, the majority of the patient population in this study was of Hispanic ethnicity. When extrapolating the current results to other populations, demographic and contextual variables within the population of interest should be considered. While we explored access to healthcare, some other social determinants of health remained unexplored. Future studies might consider marital status/family living environment, income, education level, and access to healthcare facilities that might serve as factors to explain presentation delays following hip fracture.

## 5. Conclusions

Major emergencies present challenges for patients seeking healthcare. Although patients have been showing delayed presentation after hip fracture, it does not appear to interfere with the patients' short-term outcomes. The stay-at-home orders likely lead to an increase in familism in Mexican-American families, impacting the incidence and severity of fractures in seniors. A decrease in the severity of the hip fractures may explain the higher tolerance to delays in the presentation. The observed changes in delayed presentation for hip fractures may be unique to this pandemic; thus, permanent changes in patients' behavioral patterns need to be confirmed with longer exposure times and subsequent studies. Although we propose the possibility of guided delayed treatment in future emergency events, additional studies are necessary to determine the criteria, feasibility, and sequelae of treatment delay.

## Figures and Tables

**Table 1:** Demographic characteristics of patients.

Variable	All	2019	2020	*P*-value
Gender, *n* (column %)				0.967
Males	28 (36.8)	15 (36.6)	13 (37.1)	
Females	48 (63.2)	26 (63.4)	22 (62.9)	

Ethnicity, *n* (row %)				0.942
Hispanic	61 (80.3)	33 (80.5)	28 (80.0)	
Non-Hispanic	15 (19.7)	8 (19.5)	7 (20.0)	

Age, mean (SD)	76.4 (14.9)	76.3 (15.6)	76.5 (14.3)	0.907

BMI, mean (SD)	25.4 (4.9)	25.2 (5.3)	25.5 (4.5)	0.751

BMI: body mass index.

**Table 2:** Categorical clinical characteristics of patients.

Variable *n* (column %)	All	2019	2020	*P*-value
Mode of injury				0.419
Fall	71 (93.4)	38 (92.7)	33 (94.3)	
MVC	3 (4.0)	2 (4.9)	1 (2.9)	
Other	2 (2.6)	1 (2.4)	1 (2.9)	

Type of injury				<0.001^*∗*^
D-femoral neck	15 (19.7)	10 (24.4)	5 (14.3)	
D-Inter.	22 (28.9)	18 (43.9)	5 (11.4)	
ND-femoral neck	12 (15.8)	4 (9.8)	8 (22.9)	
ND-Inter.	27 (35.5)	9 (21.9)	18 (51.4)	

Arrived at				0.412
ER	69 (90.8)	38 (88.6)	31 (90.8)	
Clinic	7 (9.2)	3 (7.3)	4 (11.4)	

Payer				0.0336^*∗*^
Medicare	56 (73.7)	32 (78.1)	24 (68.6)	
Medicaid	4 (5.3)	3 (7.3)	1 (2.9)	
Private/other	13 (17.1)	5 (12.2)	8 (22.8)	
Self-paid	3 (3.9)	1 (2.4)	2 (5.7)	

Disposition				0.013^*∗*^
Rehabilitation	43 (56.6)	19 (46.3)	24 (68.6)	
Skilled nursing	16 (21.1)	12 (29.3)	4 (11.4)	
Home/self-care	15 (19.7)	9 (21.9)	6 (17.1)	
Other	1 (1.3)	1 (2.4)	0 (0)	
Died	1 (1.3)	0 (0)	1 (2.9)	

Postsurgical complications^*∗*^				0.728
None noted	63 (82.9)	34 (83.0)	28 (80.0)	
Respiratory complications	5 (6.6)	3 (7.3)	2 (5.7)	
Cardiac complications	3 (3.9)	1 (2.4)	2 (5.7)	
Anemia	3 (3.9)	1 (2.4)	2 (5.7)	
Stroke	1 (1.3)	1 (2.4)	0	
UTI	1 (1.3)	1 (2.4)	0	

Mortality at 6 months				0.332
Died	6 (7.9)	4 (9.8)	6 (17.1)	
Unknown status	4 (5.2)	2 (4.9)	2 (5.7)	

**Table 3:** Quantitative clinical characteristics of patients.

Variables, *n* (SD)	All	2019	2020	*P*-value^+^
Delay in presentation (days)	2.6 (8.2)	0.7 (2.3)	4.8 (11.5)	**0.033** ^*∗*^
Hours from arrival to surgery	40.5 (29.0)	40.2 (29.0)	40.9 (28.9)	0.920
Hospital LOS in days	5.9 (3.2)	5.4 (2.5)	6.5 (3.7)	0.275
Number of days in ICU	0.4 (1.7)	0.1 (0.4)	0.8 (2.4)	0.152
Number of comorbidities	2.7 (1.6)	2.7 (1.6)	2.5 (1.6)	0.550

+: nonparametric test.

## Data Availability

The patients' information data used to support the findings of this study are restricted by the DHR Health Institute for Research and Development Institutional Review Board to protect patient privacy and comply with HIPPA laws. The data are available from the corresponding author, Dr. M. Serra-Torres, for researchers who meet the criteria for access to confidential data. Dr. Serra-Torres can be reached by e-mail at m.serra@dhr-rgv.com.
